# Supplementary material for: Accessing habitat suitability and connectivity for the westernmost population of Asian black bear (Ursus thibetanus gedrosianus, Blanford, 1877) based on climate changes scenarios in Iran
Source: PLoS One. 2020 Nov 18;15(11):e0242432. doi: 10.1371/journal.pone.0242432 (PMC7673494; doi:10.1371/journal.pone.0242432)
Supplement: Attachment — Submitted filename: PONE-D-20-07352.docx [file pone.0242432.s002.docx]

Ms.No.PONE-D-20-07352

The study models current and future niche of Asiatic black bear in the Iranian landscape. Perks of this study include modeling not only current but also future distribution, developing maps under several climate scenarios, and identifying potential suitable corridors. While I certainly feel as though this could be an interesting analysis, as the manuscript stands there are enough methodological and modeling concerns that would require a complete reconstruction of the analysis and manuscript. Indeed, the introduction is a bit convoluted and difficult to follow, and the entire manuscript is in need of thorough review for grammar (e.g., tense agreements), punctuation, and general continuity and flow. Of greater concern, however, are the outdated methods implemented. While I realize that this study is a primarily a model-based assessment, and as such, there is a higher bar when it comes to the appropriate methodological approaches. As they are communicated presently, I have little confidence that the best models possible were generated and doubt that they provide any additional or useful insight about the study species. Because of these methodological issues, and subsequent lack of confidence in the models produced, I have provided minimal commentary on the results and discussion sections and focus almost exclusively on methods.

Please find my detailed comments below.

Introduction.—The introduction of a manuscript should very clearly set the contextual stage for the reader. In its current form, much of the necessary clarity is lacking. Including a section that provides explicit insight into the study species, including the natural history/behavioral traits as justification for the vulnerability of the species to habitat fragmentation and climate changes would help tremendously.

Methodology.— Several concerns are raised related to the species distribution modeling such as the sample size, difficulty in understanding details of the model, potential bias in the use and distribution of pseudo absences, and the lack of any true evaluation of uncertainty due to the selection of the climate data or different GCMs scenarios.I have serious concerns about the methodology implemented, namely the fact that they are obviously not in line with current time-averaged distributional modeling best practices but also because they're not entirely clear. As it stands, I do not feel that the methods are optimized to yield reliable results and, as such, would be hesitant to draw any substantial conclusions from final models.

Following are my primary concerns.

Study species primary occurrence data quality and sampling:
- Specify: when were occurrence points collected? Why did not the authors inspect potential spatial bias? What was the sampling strategy used to split the species records into subsets for model calibration and internal evaluation? And what was the applied strategy used to sample pseudo-absence (or background) points and in which number?

Covariate selection: I have multiple issues with the covariate selection process used.
- First and foremost, I'm completely baffled as to why the authors used WorldClim data for this project. Their study species data were collected currently as far as I realized from the text, lines 253-255, while WorldClim data are climatic averages from 1970-2000. I think it would be better to select a (or better, generate your own) covariate dataset for time spans close to their collected data, for example looking at a set of bioclimatic variables from the Climatologies at high resolution for the Earth’s land surface areas (CHELSA) dataset.
- Further, as it is currently written, it appears that there was not enough consideration put into covariate selection. That is, rather than choosing covariates based on relevance to the study species, the authors simply tossed all covariates into a statistical selection process with no further discussion as to why or why not the selected variables might actually make sense relative to the study species. Lines 458-469 and Fig. 3 (d, j, g) simply present what best fit the primary species occurrence data, and are not necessarily a reflection of the covariates that are actually most important for the study species. It is also likely to result in the overfitting issue when there is an unbalance number of predicators to the species occurrences . The final occurrence dataset have to have a reasonable sample size to ensure a minimum of 5 occurrences per predictor (i.e. to avoid overfitting the models) and for being consistence with regular niche modeling protocols. Consult please the following papers:

- - Araújo, M. B., and A. T. Peterson (2012). Uses and misuses of bioclimatic envelope modeling. Ecology. https://doi.org/10.1890/11-1930.1)
- - Feng, X., D. S. Park, Y. Liang, R. Pandey, and M. Papeş (2019). Collinearity in ecological niche modeling : Confusions and challenges. 10365–10376.
- Warren, D. L., N. J. Matzke, and T. L. Iglesias (2020). Evaluating presence-only species distribution models with discrimination accuracy is uninformative for many applications.

- Future projection scenarios: On what basis was the CCSM selected? Some citation suggesting that this is appropriate for your region of interest would be ideal. Otherwise, it is highly recommended to use the outputs of a large number of GCMs under multi-emission scenarios to improve our understanding of the degree of uncertainty on projections of future species’ distribution (Goberville, E., et al. (2015). Uncertainties in the projection of species distributions related to general circulation models. Ecology and Evolution 5:1100–1116).

Model calibration region: I'm failing to see why the authors adequately did not delimitate accessible areas to the study species during the study period. Because the geographical extent of the accessible area, which depends on opportunities for and constraints on movements of the species ( Barve, et al. (2011). The crucial role of the accessible area in ecological niche modeling and species distribution modeling. Ecological Modelling 222:10) affects the outcomes of the ecological niche modelling exercise ( Anderson, et al. (2010). The effect of the extent of the study region on GIS models of species geographic distributions and estimates of niche evolution: Preliminary tests with montane rodents (genus Nephelomys) in Venezuela. Journal of Biogeography 37:1378–1393, Barve et al. 2011), the delimitation of the calibration area should reflect this issue.

Background selection: First: how many points? And, second, what was the sampling strategy? Why not generate a bias layer to ensure that background data distribution are reflective of any sampling bias within the presence data? See the following literature:

- Anderson, R.P. (2003) Real vs. artefactual absences in species distributions: tests for Oryzomys albigularis (Rodentia:Muridae) in Venezuela. Journal of Biogeography, 30, 591-605.
- Kramer-Schadt, S., Niedballa, J., Pilgrim, J.D., Schröder, B., Lindenborn, J., Reinfelder, V., Stillfried, M., Heckmann, I., Scharf, A.K., Augeri, D.M., Cheyne, S.M., Hearn, A.J., Ross, J., Macdonald, D.W., Mathai, J., Eaton, J., Marshall, A.J., Semiadi, G., Rustam, R., Bernard, H.B., Alfred, R., Samejima, H., Duckworth, J.W., Breitenmoser-Wuersten, C., Belant, J.L., Hofer, H., & Wilting, A. (2013). The importance of correcting for sampling bias in MaxEnt species distribution models.
- Fourcade, Y., Engler, J. O., Rödder, D., & Secondi, J. (2014). Mapping species distributions with MAXENT using a geographically biased sample of presence data: a performance assessment of methods for correcting sampling bias. PloS one, 9(5), e97122. <https://doi.org/10.1371/journal.pone.0097122>
- Syfert MM, Smith MJ, Coomes DA (2013) Correction: The Effects of Sampling Bias and Model Complexity on the Predictive Performance of MaxEnt Species Distribution Models. PLOS ONE 8(7): 10.1371/annotation/35be5dff-7709-4029-8cfa-f1357e5001f5. https://doi.org/10.1371/annotation/35be5dff-7709-4029-8cfa-f1357e5001f5

Modeling algorithm: Why was only a single statistical algorithm explored? Yes, Maxent is a fantastic tool, but numerous studies indicate that there is no "one size fits all" when it comes to algorithm selection in distributional modeling. I highly recommend that the authors review the article "No silver bullets in correlative ecological niche modelling: insights from testing among many potential algorithms for niche estimation" by Qiao et al. (2015), Methods in Ecology in Evolution 6(10):1126-1136.

Model selection: (Line 364) Why use ROC? There well-documented issues with ROC (See: Peterson et. al. 2008. Rethinking receiver operating characteristic analysis applications in ecological niche modeling. Ecological Modelling 213:63-72).

Model thresholding: Why did not the authors mention what a specific emission threshold apply ? If the authors were using open access (e.g., GBIF) data, then I would expect a 5% threshold to be about right. But, considering the authors are using data in which they should have much greater confidence, a low threshold seems particularly appropriate. Perhaps 1% would be more suitable for the data at hand?

Model uncertainty: It will be useful to control model variations by generating final models using the selected parameterizations and performing multi-replications by bootstrap or cross-validation for each scenario. It will help the authors to compare differences in potential changes in future projections (Table 4) and also test whether the total amount of suitable area significantly increased or decreased between different independent climatic scenarios.

Discussion.—As the authors use their distributional models to draw conclusions about the potential connections’ pathways and also potential changes in black bear through time, they would do well to not forget that niche models are a projection of the environmental niche onto the geographic landscape. In other words, model predictions are only a single facet of a suite of factors that determine a species' distribution that include but are not limited to the availability of suitable habitat, accessibility, and other abiotic factors (e.g., landscape geometry).
