# Supplementary material for: Accessing habitat suitability and connectivity for the westernmost population of Asian black bear (Ursus thibetanus gedrosianus, Blanford, 1877) based on climate changes scenarios in Iran
Source: PLoS One. 2020 Nov 18;15(11):e0242432. doi: 10.1371/journal.pone.0242432 (PMC7673494; doi:10.1371/journal.pone.0242432)
Supplement: Attachment — Submitted filename: Dear editor.docx [file pone.0242432.s003.docx]

**Dear editor**

Please accept our apology for an honest mistake in the paper title. I’m writing to you to let you know that title of the manuscript was changed in one word and correct title is:

“Accessing Habitat Suitability and Connectivity for the Westernmost Population of Asian Black Bear (*Ursus thibetanus gedrosianus*, Blanford, 1877) based on Climate Changes Scenarios in Iran”

This change is also applied in the revised version of the manuscript

By considering the Email from Mrs. Anna Mental

1- I should note that all map images were created with data owned/collected by the authors.

2- Supporting information including SHP file of the protected area, present/absent points and boundary of the study area were added to the folder with title of “supporting information”.

Best regards

Maryam Morovati
