# Supplementary material for: Accessing habitat suitability and connectivity for the westernmost population of Asian black bear (Ursus thibetanus gedrosianus, Blanford, 1877) based on climate changes scenarios in Iran
Source: PLoS One. 2020 Nov 18;15(11):e0242432. doi: 10.1371/journal.pone.0242432 (PMC7673494; doi:10.1371/journal.pone.0242432)
Supplement: Attachment — Submitted filename: Ms.No.PONE-D-20-07352_R2.docx [file pone.0242432.s004.docx]

Ms.No.PONE-D-20-07352

There were several parts of the manuscript (MS) that were confusing and unfocused despite reviewer#2 highlighted several major issues, especially those pertaining to methodological and modeling concerns including the delimitation of ‘model calibration region’ and ‘Model uncertainty’. As the MS stands, much of the confusion seems to come from methods and results sections yet because the whole paper did not comprehensively follow reviewer’s comments. Some of my specific comments below may help, but in general, the authors should try to improve readability and clarity by thinking about the organization of the paper and the logical transitional flow between ideas.

SPECIFIC/MINOR ISSUES

Line 25: change ‘communications’ with ‘connectivity’

Line 28: remove ‘For this purpose’

Line 28: change ‘3 provinces’ to ‘provinces’

Line 29: change ‘Sistan and Baluchestan’ to ‘Sistan-Baluchestan’. Follow this afterwards throughout the text.

Line 29: remove ‘and then, corrected in terms of spatial autocorrelation’

Lines 30-31: delete the abbreviations of modelling techniques

Line 30: change ‘,3 machine learning models of’ to ‘ and 3 machine learning models including’

Line 31: Ensemble model is not itself a unique modelling algorithm, it is just an approach to combine the results of formal habitat modelling algorithms. So, the authors should address how the ensemble model was undertaken to integrate the main habitat models as a separate sentence.

Lines 32-34: I am not sure that I could understand well what the authors mean by how they generated the background points to undertake the habitat modelling. It would better not mention it here and go through to explain it in depth in the appropriate part of the MS.

Line 36: remove ‘,which have the best response for Iran,’

Lines 39-40: rephrase as: ‘The true skill statistic was employed to convert the continuous suitability layers to binary suitable/unsuitable range maps to assess the effectiveness of the protected areas in the coverage of suitable habitats for the species.’ It should point out why the authors chose this evaluation metric to dichotomize the habitat suitability, not here but the next parts of the MS.

Lines 40-41: better to rephrase as: ‘The random forest method was also used to predict the relationships between the presence probability of the species and the environmental predicators.’ But, it should be highlighted in the other sections of the MS not here the reasons behind selecting this model to evaluate the species response curves to the predictors.

Line 42: To be specific. which model(s)? Did all models perform well in discriminating suitable and unsuitable areas?

Line 42: what does mean by ‘successful in the implementation’?

Line 43: be specific in what bio17, … stands for? Then delete the words of bio17, … here.

Lines 43-44: clarify what the authors mean by ‘high’ and ‘extreme’?

Line 45: To which habitat modelling techniques were the area of suitable habitats shown in parentheses associated?

Line 46: replace ‘higher’ with ‘larger’ and ‘the current’ with ‘the current distribution’.

Line 46: ‘all the models run under…’. I am completely confused about why the authors applied the ensemble model regardless they are interested in addressing the changes of the species habitat suitability through the application of every single model? by considering that the main idea behind using the ensemble modelling is to deal with the uncertainties inherited from each modelling algorithm.

Line 49: ‘Province’ should be province. Do this correction afterwards where it is needed.

Line 52: what does mean by ‘have higher quality habitat connections’?

Introduction:

Line 64: ? 4 written as superscript to ‘wild animals’

Line 72: ‘ in which case …’. I am unable to follow what the authors intend to say. It does need to be rephrased to make it more clarification.

Line 76: change ‘the surest way’ to ‘the reliable way’

Line 83: ‘the manager’ to ‘a manager’

Line 87: what does mean by ‘frequency’?

Line 92: explain how human pressure can contribute to the species vulnerability?

Line 95: perhaps you mean ‘reaches’ by ‘reches’?

Line 96-97: needs a citation!

Line 99: ‘IUCN classification’ to ‘IUCN red list’

Lines 100-101: give a reference; however if the authors expect the negative consequences of climate changes on the species, they need to rewrite the sentence by the consideration of the possibility of incidence.

Lines 103-108: the facts that have pointed out here is really important and then it needs to get involve the reliable references.

Materials and methods:

Line 115: ‘ presence points’ better to change ‘species occurrences’

Line 121: replace ‘symptoms’ with ‘signs’.

Line 123: replace ‘navigating’ with ‘covering’

Figure 1:

- Limit the numbers of longitudinal and latitudinal coordinates around the box.
- Steak to the instructions of the figures’ preparation guidelines of the journal to meet whatever needs to be considered to draw the figures more transparent.
- Explain what you mean by the red crooked line and the word of ‘locality’ inset in the map?
- Generally, figure should represent more visible the main protected area, instead it has emphasised on marginal components. So, it is highly recommended to be provided more informative base map.

Lines 142-143: rewrite as distances from gardens, main roads, streams (waterways), and human

settlements, …

lines 156-157: how did the authors come into that a 3×3 filter is the best spatial resolution to address the habitat fragmentation?

Line 161: replace ‘of’ to ‘including’

Lines 165-166: I think the 2.6 RCP is not related to the mild seanarios?!

Lines 166-167: delete ‘Various GCM models …’

Table 2:

- The caption should reflect what the study aimed at using the environmental variables.
- What was meant by ‘low’ in the third column?
- In the fourth column, what do the authors want to say by ‘Current-RCP2.6-RCP8.5’? Does not it that RCPs are an indication on how the green house gases’ concentration will be in the future? So, how did you integrated those trends with the current time?
- You can simply rescale the values of the bioclimatic variables corresponding to the temperature in the third column to make them real temperatures.

Line 175: I would prefer to go with distribution instead ‘dispersion’ throughout the text. Please consider this in wherever you encounter with the dispersion in the MS.

Line 176: change ‘of Maxent’ to ‘including Maxent’.

Line 176-177: it would better go for ‘To model the species distribution’ instead of ‘In the implementation of the models’

Line 190: Why did the authors consider only topographic heterogeneity to inspect spatially auto-correlated occurrence points despite their study is aiming at the assessment of how environmental variables (vegetation, climatic, and topographic covariates) can predict the species habitat suitability. Meanwhile, what methods did they apply to deal with this issue? For example it is strongly recommended to look at the global and local Moran coefficients (Boots, B., 2002. Local measures of spatial association. EcoScience9, 168–176).

Line 204-205: How was the ensemble map created?

Line 207-208: I am not sure why the authors intend to relate the species adaptation to climate change to the current protected areas (PAs) network. Are they sure the current PAs will sufficiently meet the future species climate requirements?. Specifically, the authors need to explicitly outline the biological justifications that they aim to address through this investigation.

Line 214: change ‘circuitscape’ to ‘Circuitscape’

Line 214-218: This section would benefit from some more references to back up and provide further detail.
Line 219: what is ‘The cost or Friction map’? would be added a citation! In addition, the authors sharply jumped into this paragraph without a logical transition from the later paragraph. It would be better rephrase these sections to follow the authors’ thoughts more easily.

Line 221: What is a difference between the cost and resistance maps?

Line 225: Add reference(s) to ‘or as the boundary of protected areas.’

Line 225: What does mean by ‘focal nodes’?

Lines 212-229: The use of ‘electronic circuit theory’ in the Methods needs more development in terms of recasting in a way that can be interpreted ecologically. Currently it reads very much as having applied a "black box" with foreign (to wildlife research at least) terms such as "current flow" and "resistance". Can these be recast as more accessible descriptions for the reader?

Line 231: I am really confused why the authors did employ a modeling technique (random forest) to see the contribution importance of the environmental variables in the species occurrence probability that was not included in an array of the applied modeling methods i.e. GLM, Maxent, SVM, and BP-ANN (lines 175-176) which were used to predict the species distribution.

Lines 232-242: This section can be shortened more logically by adding more relevant refrence(s) to facilitate reader to follow the authors’ thoughts.

Line 241-242: This is extremely good to see the authors are using more applicable scientific program (R) here, by considering this point, I am really baffling why they did not use the potential of R program which is publically free available through more compressive species distribution analyzing packages to take into account the latest developments in species distribution modeling. I truly believe that this study will have meaningful contributions to the species ecology and conservation if the authors would polish their analyses in R!.
Line 247 and Table 3: It is very interesting that I see the authors are using the significant metric (p-value) to address their findings. But it would be really useful to be added more descriptions in the methodology part of the MS how they did statistically compare the models performances using the evaluation metrics. This analysis would be more reproducible if the authors back their analysis given a reference(s). It is urgently needed to add more clarification on it since this is very important point.

Line 256: ‘the different RCP 2.6 scenarios’: It is not more clear. How many scenarios are available for RCP 2.6.? I assume that there is just one RCP 2.6!

Line 258: ‘Under the RCP8.5 scenario, the habitat suitability declines in the northern parts…’. How do the authors come to this finding? It is worthy to be specific here by giving more information where and how many suitable patches will be lost under this scenario? AS the ms stands (lines 258-260), it is not scientifically informative to visually interpret the change trends of the species habitat suitability.

Lines 265-266: How did the authors go straight to this point? Did they know where the suitable/unsuitable areas occur already to draw this interpretation?

Table 4: What does mean by greater than for the thresholds of the model in the second column? Did the authors use these threshold levels to binaries the continuous suitability maps? If so, how? And also for what metrics (TSS, AUC, Kappa, …) was these levels calculated? It is really important to be awareness on uncertainties inherits in developing the models. So, the authors are strangely recommended to iterate each model several times (e.g. 30 times) to ensure that the results are expressed statistically meaningful in terms of the confidence intervals when it goes to consider the model performances using the elevation metrics.

Lines 277 and 278: What do the authors mean by the similarities? On base of what quantity metric(s) do they go for through the more similarities’ claim?

-The terms ‘current flow’ (line 227) and ‘amps’ (line 299) seem a misnomer. Can you express in a more ecological manner that makes more sense to the reader? For example, the term ‘connectivity’ has an ecological meaning, but ‘current flow’ doesn’t at this stage.

Line 323: What do bio19, 17, and 7 stand for? Here it is good to define these abbreviations. How do species response to these climatic variables. Creating response curves for them would add more value to the study.

Line 327: Capitalize ‘random’

-table 6: Remove unnecessary horizontal and vertical lines. What is ‘Peasant’? maybe you mean ‘present’. Uncertainty on these findings could be controlled if the authors performed multi replicates (e.g. 30 times) of their model as I mentioned before in developing species distribution modeling (please also note the comments on Table 4).

\# General comments on figures: The figures are predominantly drawn in a way making it hard to interpret the main points what the authors would intend to present. Generally, what is highlighted in each figure exactly is not the main goal of the study but some main points that are needed to be more visible and clearer; unfortunately, they did not take into consideration for them.
